# The effects of a thermogenic supplement on metabolic and hemodynamic variables and subjective mood states

**DOI:** 10.1080/15502783.2023.2185538

**Published:** 2023-03-02

**Authors:** Jessica M. Prather, Christine M. Florez, Amie Vargas, Bella Soto, Abby Harrison, Darryn Willoughby, Grant Tinsley, Lem Taylor

**Affiliations:** a University of Mary Hardin-Baylor, Human Performance Lab, School of Exercise and Sport Science, Belton, TX, USA; b Texas Tech University, Energy Balance & Body Composition Lab; Department of Kinesiology & Sport Management, Lubbock, TX, USA

**Keywords:** Thermogenic supplement, Fat burners, Supplements, Multi-ingredient

## Abstract

**Background:**

Thermogenic supplements are widely used in the general population to support attempted fat loss; however, the efficacy and safety of these supplements are questioned.

**Purpose:**

To determine whether a thermogenic supplement affects metabolic rate, hemodynamic responses, and mood states.

**Methods:**

In a randomized double-blind crossover design, 23 females (22.2 ± 3.5 years; 164.8 ± 6.4 cm; 73.5 ± 6.9 kg) who were moderate caffeine consumers (<150 mg/day) reported to the lab after a 12 h fast for baseline assessments of resting energy expenditure (REE) via indirect calorimetry, heart rate (HR), blood pressure (SBP and DBP), blood variables, and hunger, satiety, and mood states. Thereafter, subjects ingested the assigned treatment (active treatment containing caffeine, micronutrients, and phytochemicals [TR] or placebo [PL]). All variables were reassessed at 30-, 60-, 120-, and 180 min post-ingestion. Subjects repeated the same protocol with ingestion of the opposite treatment on a separate day. All data were analyzed using a 2 × 5 ANOVA with repeated measures and significance was accepted a priori at *p* < 0.05.

**Results:**

In the TR group, mean increases in REE of 121 to 166 kcal/d were observed at 30-, 60-, and 180 min post-ingestion (*p* < 0.01 for all). PL group mean decreases in REE of 72 to 91 kcal/day were observed at 60-, 120-, and 180 min (*p* < 0.05 for all). Respiratory quotient decreased at 120 and 180 min in both treatments. Slight increases in SBP of 3–4 mmHg were observed at 30, 120, and 180 min (*p* < 0.05 for all) post-ingestion of TR, while no effects were observed for DBP. Observed increases in SBP were within normal blood pressure ranges. TR decreased subjective fatigue with no other significant changes in mood states. Glycerol was maintained in TR, while there was a decrease at 30, 60, and 180 min (*p* < 0.05 for all) post-ingestion of PLA. Free fatty acids increased in TR at 60 and 180 min (*p* < 0.05) post-ingestion as well as a significant difference between treatments at 30 min post-ingestion indicating greater circulating free fatty acids levels in TR vs. PL (*p* < 0.01).

**Conclusion:**

These findings indicate that ingestion of a specific thermogenic supplement formulation produces a sustained increase in metabolic rate and caloric expenditure and reduces fatigue over 3 h without producing adverse hemodynamic responses.

## Introduction

Thermogenic supplements are widely used in the general population to support attempted fat loss; however, the efficacy and safety of the variety of ingredient blends in such supplements are questioned. Thermogenic products can stimulate metabolic rate and the production of heat, which is why supplements that claim to acutely increase energy expenditure or fat metabolism are commonly referred to as fat burners.

Caffeine is often the main ingredient in commercially available thermogenic supplements due to its ability to increase resting energy expenditure (REE) as well as augment fat oxidation and fatty acid turnover both at rest [1] and during exercise [2]. The combination of caffeine with a variety of additional herbal ingredients, however, is claimed to have a greater benefit on metabolism [3–7].

Compounds that mimic the activity of the sympathetic nervous system (SNS) are known as sympathomimetic because they stimulate thermogenesis and fat oxidation similarly to true SNS activity [8], and this stimulatory effect is a main mechanism of action for many of the active ingredients in thermogenic supplements. Activation of the SNS has been shown to stimulate lipolysis, suppress hunger, and increase satiety, as well as produce a thermogenic effect; however, these signals are dependent on the accumulation of cyclic-adenosine monophosphate (cAMP), which is inhibited by phosphodiesterase [9]. Caffeine assists in the stimulation of the SNS by binding to adenosine receptors, thus decreasing the reduction of excitability, and inhibiting PDE, which ultimately leads to an accumulation of cAMP.

Several other sympathomimetic compounds found in thermogenic supplements are believed to augment the accumulation of cAMP by stimulating β-2 and β-3 adrenergic receptors. When caffeine is combined with these other ingredients, the accumulation of cAMP, and subsequently increases in circulating epinephrine and free fatty acids [3], results in an increasingly excitatory environment for cells [8].

Stimulant-based supplements often raise concerns for the potential negative impact on hemodynamic variables such as heart rate (HR) and blood pressure (BP). Elevations in HR and BP have been reported in some studies involving thermogenic supplements containing caffeine [10], while others provide evidence to support the contention that ingestion of caffeine-containing supplements does not negatively impact the same variables [7].

Of the available literature on thermogenic supplements and their impact on metabolic rate and hemodynamic responses, the outcomes seem to be contradictory in the magnitude at which the effect is observed. Furthermore, the safety and efficacy of specific, commercially available multi-ingredient supplements must be evaluated. Therefore, the purpose of this study was to determine whether a thermogenic supplement affects metabolic rate, hemodynamic responses, and mood states, and to what extent these responses were seen.

## Materials and methods

### Participants

The participants in this study were healthy female college students (22.2 ± 3.5 years; 164.8 ± 6.4 cm; 73.5 ± 6.9 kg) between the ages of 18–40 with a body mass index (BMI) between 25 and 32.5 kg*m^2^ and were selected from a pool of volunteers. Approval for this study protocol was granted by the University of Mary Hardin-Baylor Institutional Review Board. After the participants were successfully recruited, they were addressed and informed in the Human Performance Lab (HPL) at the University of Mary Hardin-Baylor of the rationale and purpose of this study and their right to refuse or discontinue participation at any given time throughout the study. Consenting participants were then scheduled for a familiarization session where they provided their written and verbal consent and completed a medical history form, general health and information screening form, and a caffeine consumption questionnaire. To participate in the study, participants needed to be healthy and free of any cardiovascular, pulmonary, or metabolic disease. Participants were excluded if they consumed more than 150 mg of caffeine before 12:00 pm daily, or as a result of any allergy or sensitivity to the supplement ingredients.

### Experimental design

**Figure 1.**
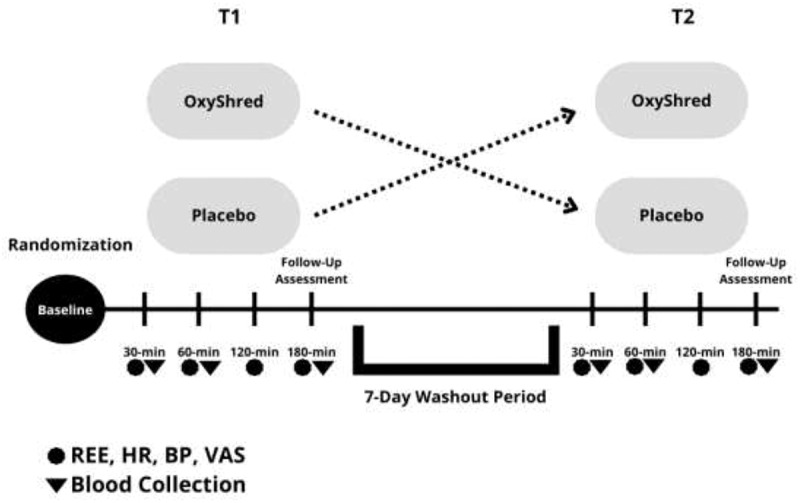
Overview of testing sessions.

This study utilized a randomized, double-blind, placebo-controlled crossover design is outlined in Figure 1. Testing for this study was completed during two sessions that took place on two separate days, with at least 7 days between each visit. Testing was in a climate-controlled room with the lights dimmed during testing. The majority of testing sessions in the HPL were scheduled and occurred in the morning hours before 11:00 am, and one participant completed testing in the early afternoon due to scheduling conflicts.

After abstaining from exercise for 24 h prior to the testing session, participants were instructed to report to the HPL following a 12 h fast. The height of the participants was measured using a stadiometer (Seca 264 Digital Stationary Stadiometer, Hamburg, Germany), and weight was measured using a body composition scale (Tanita Body Composition Analyzer, model TBF-310, Arlington Heights, Illinois, USA) to assess BMI. All REE measurements were assessed using the TrueOne 2400 Metabolic Measurement System (ParvoMedics, Sandy, Utah, USA), which was calibrated daily approximately 30 min before the start of the testing session.

REE was measured by placing a clear, hard plastic hood with a clear plastic drape attached to the metabolic cart over the participant’s torso to measure oxygen inspiration and carbon dioxide expiration. Blood pressure was assessed using a digital blood pressure monitor (Omron Professional Intellisense Blood Pressure Monitor, model HEM-907XL, Kyoto, Japan). Participants were then prepared for baseline assessments of REE and associated variables [inspired oxygen (VO_2_), expired carbon dioxide (VCO_2_), and respiratory quotient (RQ)] via indirect calorimetry, HR, systolic and diastolic blood pressure (SBP and DBP), as well as subjective mood states via a visual analog scale (VAS), and lastly a venous blood sample was obtained. Thereafter, participants ingested their randomly assigned treatment of either the active treatment (TR), or placebo (PL), which was determined by using an online random number generator (random.org/lists). Following ingestion, participants remained in the lab in a rested state for the duration of the 3 h post-ingestion assessment period. REE, HR, BP, and mood states were reassessed at 30, 60, 120, and 180 min post-ingestion of the treatment, while blood was collected again at 30, 60, and 180 min post-ingestion. Participants also completed a follow-up questionnaire reporting any adverse events that may have occurred upon completion of each testing appointment. Participants completed the same protocol with ingestion of the opposite treatment at the second and final testing session on a separate day at least 7 days after the first testing session, following the same pre-testing instructions.

### Blood collection

Fasted blood samples were collected using an intravenous (IV) catheter to assess glycerol and free fatty acids. Vials of blood were prepared, labeled, and collected using standard phlebotomy techniques according to the requirements of each assay. A Serum Separator Tube (SST) and a dipotassium ethylenediaminetetraacetic acid tube (K_2_ EDTA) were used to collect blood samples at baseline. The SST was centrifuged for 15 min after sitting for 30 min at room temperature. Thereafter, 1 mL of serum was extracted using micropipettes and deposited into a properly labeled transport tube and stored at -80°F until they were sent off for analysis on the same day. The K_2_ EDTA tubes were chilled on ice for 10 min before being centrifuged for 15 min. Serum was extracted using micropipettes and deposited into two properly labeled microtubes. A K_2_ EDTA tube was used at each of the 30, 60, and 180 min post-ingestion time points to collect blood and were also chilled on ice for 10 min before being centrifuged for 15 min and the serum extracted and deposited into two properly labeled microtubes.

Serum samples collected in SST were sent to Quest Diagnostics (Irving, TX) to be assayed for free fatty acids, and serum samples in the microtubes for glycerol were stored at -80°F until they were sent off for analysis. Duplicate undiluted serum samples were analyzed using a commercially available fluorometric-free glycerol assay kit (Cell Biolabs; San Diego, CA). After following standard assay procedures, standards, controls, and samples were analyzed using a fluorometric plate reader (Biotek Synergy 2; Santa Clara, CA) with an excitation wavelength of 540 nm and an emission wavelength of 590 nm. Unknown sample concentrations were calculated from the standard curve. All samples were analyzed on the same day to minimize variation, and the intraassay coefficient of variability (CV) was tested as <8%.

### Supplement

The thermogenic supplement treatment and placebo were in powder form with uniform scoop sizes and dissolved in 300 mL of cold water. Lab staff prepared the powder and water mixture to mix appropriately and observed the participants’ consumption of the treatments, which had to be completed in <5 min. The ingredients in the active treatment, which contains 150 mg of caffeine (OxyShred Thermogenic Fat Burner, EHP Labs, Salt Lake City, Utah, USA) are presented in Table 1, while the placebo contained only inactive ingredients (gum Arabic, citric acid, malic acid, NAT Watermelon Type, NAT bitter blocker, sucralose, silicon dioxide, calcium silicate, beet color powder). Treatment and placebo powders were blinded for taste, texture, and appearance, produced by the manufacturer, and arrived in blinded containers. All containers were kept at room temperature in a cool and dry location. The treatment was given to the participants after completion of all baseline testing and questionnaires.

**Table 1.** EHP Labs OxyShred thermogenic fat burner ingredients list

OxyShred (one serving)	Amount/serving	% DV
Calories	5	
Total carbohydrate	1.0 g	<1
Dietary fiber	0.2 g	4*
Vitamin C	173 mg	193
Thiamin	0.56 mg	46
Riboflavin	0.78 mg	60
Niacin	20 mg	123
Vitamin B6	0.98 mg	58
Vitamin B12	0.9 mcg	38
Pantothenic acid	1.7 mg	34
Chromium picolinate	10 mcg	3
Fat burning matrix Acetyl L-carnitine HCl, *Garcinia cambogi*a fruit extract (60% hydroxycitric acid), conjugated linoleic acid (CLA), grapefruit seed extract 4:1, raspberry ketones (from raspberry fruit extract), *Mangifera indica* seed extract, bitter orange fruit extract, green coffee bean extract (50% chlorogenic acid), olive leaf extract (10% oleuropein), guggul extract powder, chromium picolinate	2003 mg	
Immunity booster & prebiotic complex L-glutamine, inulin fiber, vitamin c (ascorbic acid)	625 mg	
Mood enhancer matrix L-tyrosine, taurine, caffeine anhydrous (150 mg), *Huperzia serrata* whole herb extract (Huperzine A)	851 mg	
Full B vitamin spectrum Niacinamide (niacin), calcium pantothenate (pantothenic acid), pyridoxine HCl (vitamin B6), riboflavin (vitamin B2), thiamine mononitrate (vitamin B1), cyanocobalamin (vitamin B12)	24.59 mg	

### Statistical analysis

Participant characteristics (age, height, weight) were analyzed using descriptive statistics. Normality and sphericity of these data were assessed using the Shapiro–Wilk test and Mauchly’s test. Metabolic, hemodynamic, and subjective mood state variables were analyzed using a 2 × 5 analysis of variance (ANOVA) with repeated measures and a Bonferroni post-hoc test. Blood variables were analyzed using a 2 × 4 ANOVA with repeated measures. No analyses were performed for the caffeine consumption questionnaire as it was used solely for determining if the participant met the entrance criteria. All statistical analyses were performed using IBM SPSS Statistics 25 (IBM, Armonk, NY) or R (v. 4.1.2). Data are presented as means ± standard deviation, and statistical significance were set a priori at *p* ≤ 0.05.

## Results

### REE, VCO_2_, VO_2_, RQ

**Figure 2.**
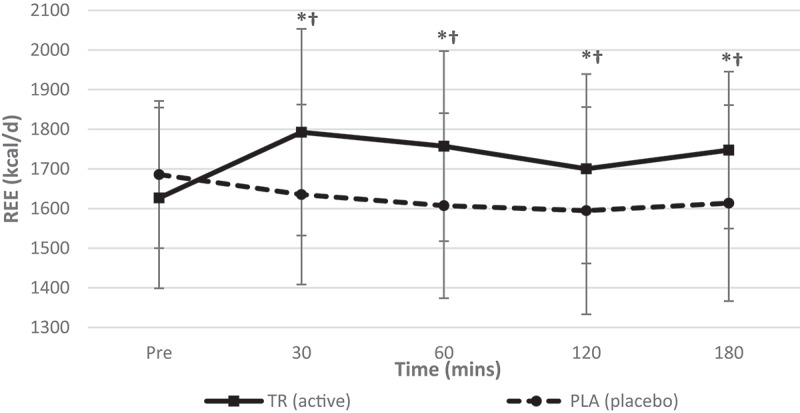
Resting energy expenditure over time. A significant interaction (condition*time) and significant main effects for time and condition were observed for REE. TR ingestion increased REE from baseline to 30, 60, 120, and 180- min while REE decreased in PLA from baseline to at all time points post-ingestion. There was also a significant main effect for condition at 30, 60, 120, and 180 min post ingestion (condition: TR = active; PLA = placebo). *Denotes statistical significance at *p* < 0.05 for differences from baseline to each timepoint; †denotes statistical significance at *p* < 0.05 for differences between conditions.

A significant condition*time interaction for REE was observed (*F* = 10.172, *p* = 0.000, η_p_^2^ = 0.316). Additionally, significant main effects for time (*F* = 3.713, *p* = 0.008, η_p_^2^ = 0.144) and condition (*F* = 28.600, *p* = 0.000, η_p_^2^ = 0.565) were observed (Figure 2). Follow-up testing with a paired samples *t*-test revealed a significant increase in REE after ingesting TR from baseline to 30, 60, 120, and 180 min (*p* < 0.001, *p* = 0.001, *p* = 0.049, *p* = 0.001, respectively), and significant decreases in REE after ingesting PLA from baseline to 30, 60, 120, and 180 min (*p* = 0.042, *p* = 0.001, *p* = 0.007, *p* = 0.02). There were also significant differences between conditions at 30, 60, 120, and 180 min post ingestion (*p* = 0.000, *p* = 0.000, *p* = 0.011, *p* = 0.000, respectively) in which TR was significantly higher (Table 2).

**Table 2.** Descriptive data for all time points is included for hemodynamic, metabolic measures for TR and PL (data presented as means ± standard deviations).

	Pre			30 min		60 min	
	TR	PL		TR	PL	TR	PL
Heart Rate (Beats/min)	60.8 ± 9.3	61.0 ± 12.3		56.0 ± 8.7	59 ± 9.9	58.1 ± 8.8	59.4 ± 9.9
Systolic Blood Pressure (mmHg)	108 ± 10	108 ± 9		112 ± 9	108 ± 12	111 ± 9	105 ± 10
Diastolic Blood Pressure (mmHg)	67 ± 6	68 ± 8		70 ± 7	71 ± 21	70 ± 6	67 ± 8
REE (kcals/d)	1626.8 ± 228	1685.7 ± 185.8		1792.7 ± 260.8	1635.3 ± 226.8	1757.4 ± 239.5	1607.2 ± 233.4
VO2 (mL/min)	240.2 ± 35.1	249.1 ± 29.6		264.2 ± 41.4	241.1 ± 34.8	259.1 ± 36.7	238.9 ± 35.9
VCO2 (mL/min)	176.6 ± 24.7	179.7 ± 21.5		195.3 ± 26.2	177.7 ± 24.5	189.3 ± 26.2	169.4 ± 24.7
RQ (VCO2/VO2)	0.7413 ± 0.08	0.7265 ± 0.09		0.74830 ± 0.09	0.7439 ± 0.08	0.7348 ± 0.07	0.7126 ± 0.07
	120 min				180 min		
	TR		PL		TR		PL
Heart rate (beats/min)	58.6 ± 9.5		61.0 ± 10.1		58.1 ± 9.7		61.9 ± 12.9
Systolic blood pressure (mmHg)	112 ± 10		105 ± 8		112 ± 10		108 ± 11
Diastolic blood pressure (mmHg)	71 ± 8		68 ± 6		70 ± 6		68 ± 9
REE (kcal/day)	1700.4 ± 239.1		1594.7 ± 261.4		1747.5 ± 197.9		1613.7 ± 247.1
VO_2_ (mL/min)	252.7 ± 38.2		236.8 ± 40.4		259.5 ± 32.9		239.5 ± 37.5
VCO_2_ (mL/min)	178.6 ± 22.2		167.0 ± 25.3		183.8 ± 18.8		169.5 ± 26.0
RQ (VCO_2_/VO_2_)	0.7126 ± 0.08		0.7109 ± 0.07		0.7091 ± 0.08		0.7113 ± 0.07

**Figure 3.**
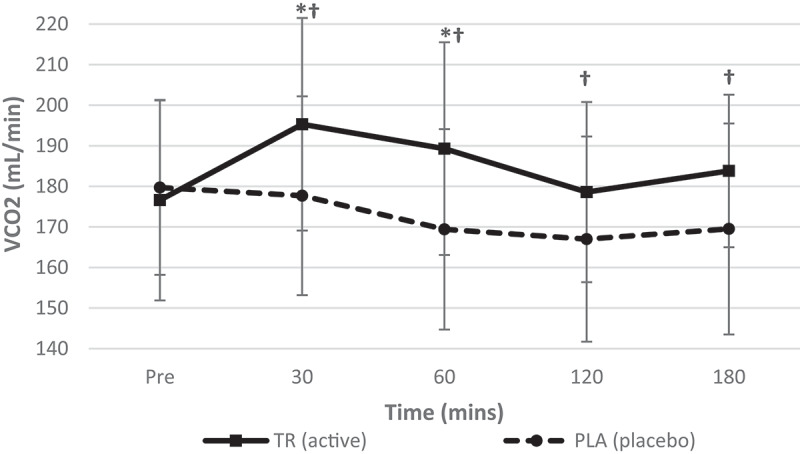
Volume of expired carbon dioxide over time. A significant interaction (condition*time) and a significant main effect for time was observed for VCO_2_. TR ingestion increased VCO_2_ from baseline to 30 and 60 min post-ingestion while VCO_2_ was significantly higher in TR than PLA at all time points post-ingestion (condition: TR = active; PLA = placebo). *Denotes statistical significance at *p* < 0.05 for differences from baseline to each timepoint; †denotes statistical significance at *p* < 0.05 for differences between conditions).

Similarly, a significant condition*time interaction for expired carbon dioxide (Figure 3) was observed (*F* = 4.642, *p* = 0.002, η_p_^2^ = 0.174), as well as significant main effects for time (*F* = 5.550, *p* = 0.000, η_p_^2^ = 0.201) and condition (*F* = 19.825, *p* = 0.000, η_p_^2^ = 0.474). Post-hoc testing revealed an increase in VCO_2_ from baseline to 30 (*p* = 0.001) and 60 (*p* = 0.016) min post-ingestion in TR as well as a significant difference between conditions at 30, 60, 120, and 180 min (*p* = 0.001, *p* = 0.001, *p* = 0.020, *p* = 0.005, respectively).

**Figure 4.**
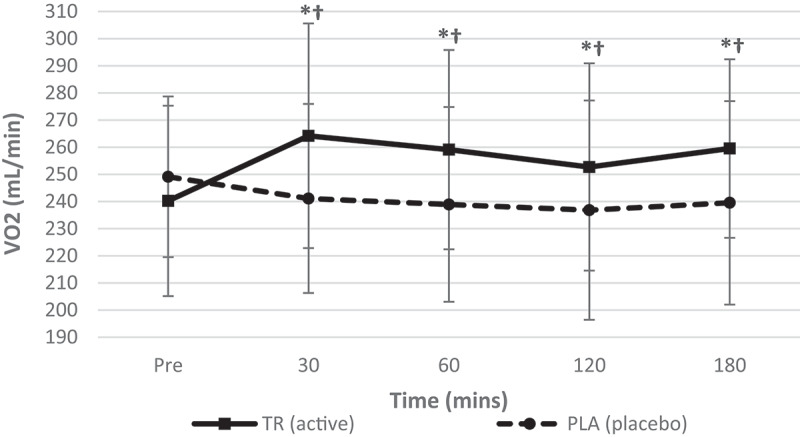
Volume of inspired oxygen over time. A significant interaction (condition*time) and significant main effect for time were observed for VO_2_. TR ingestion increased VO_2_ from baseline to 30, 60, 120, and 180 min post-ingestion. There were also significant differences between conditions at all timepoints post-ingestion (condition: TR = active; PLA = placebo). *Denotes statistical significance at *p* < 0.05 for differences from baseline to each timepoint; †denotes statistical significance at *p* < 0.05 for differences between conditions.

A significant condition*time interaction (*F* = 10.926, *p* = 0.000, η_p_^2^ = 0.332) was observed for oxygen consumption (VO_2_). Additionally, significant main effects for time (*F* = 3.103, *p* = 0.019, η_p_^2^ = 0.124) and condition (*F* = 24.293, *p* = 0.000, η_p_^2^ = 0.525) were observed (Figure 4). Post-hoc testing revealed significant increases from baseline to 30, 60, 120, and 180 min (*p* < 0.001, *p* < 0.001, *p* = 0.023, *p* < 0.001, respectively) and significant differences were seen between conditions at 30, 60, 120, and 180 min post-ingestion (*p* < 0.001, *p* < 0.001, *p* = 0.009, *p* < 0.001, respectively) following the ingestion of TR.

**Figure 5.**
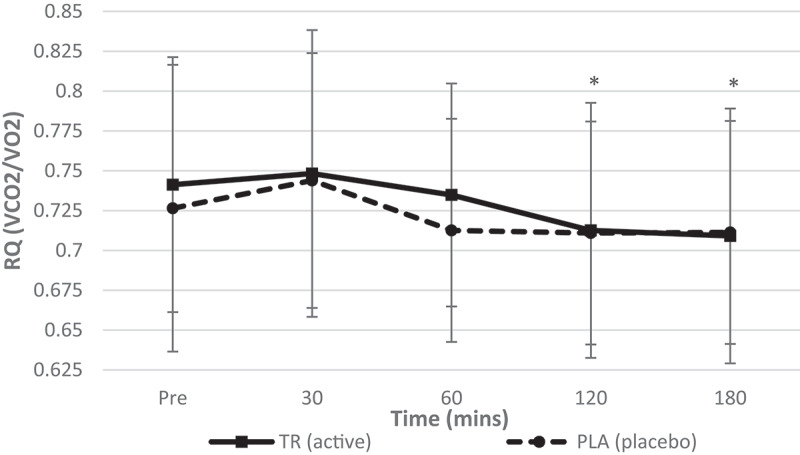
Respiratory quotient over time. No significant interaction (condition*time) was observed for RQ. A significant main effect of time, but no main effect for condition, was observed for RQ. An observed decreased in RQ for both groups was seen at 120 and 180 min post ingestion (condition: TR = active; PLA = placebo). *Denotes statistical significance at *p* < 0.05 for differences from baseline to each timepoint.

No condition*time interaction (*F* = 0.844, *p* = 0.495, η_p_^2^ = 0.037) was observed for RQ (Figure 5). A significant main effect for time (*F* = 8.757, *p* = 0.000, η_p_^2^ = 0.285) but not condition (*F* = 0.701, *p* = 0.412, η_p_^2^ = 0.031) was observed for RQ. A paired samples *t*-test revealed a decrease in RQ from baseline to 120 (*p* = 0.002) and 180 min (*p* = 0.029) post ingestion.

### Hemodynamic responses

**Figure 6.**
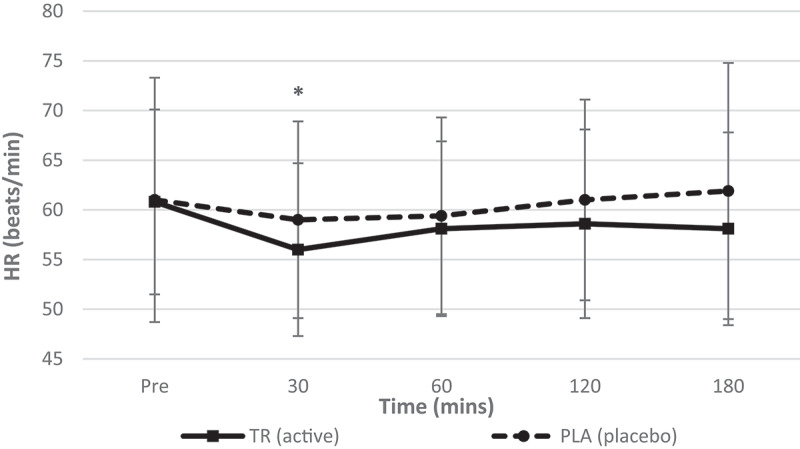
Heart rate over time. No interaction (condition*time) was observed. A significant main effect of time was observed, with follow-up indicating a decrease in heart rate at 30 min post ingestion (condition: TR = active; PLA = placebo. *Denotes statistical significance at *p* < 0.05 for differences from baseline to each timepoint.

Analysis indicated no significant condition*time interaction (*F* = 0.890, *p* = 0.153, η_p_^2^ = 0.041) was observed for HR while significant main effects for time (*F* = 2.512, *p* = 0.048, η_p_^2^ = 0.107) and condition (*F* = 4.501, *p* = 0.046, η_p_^2^ = 0.176) were observed (Figure 6).

**Figure 7.**
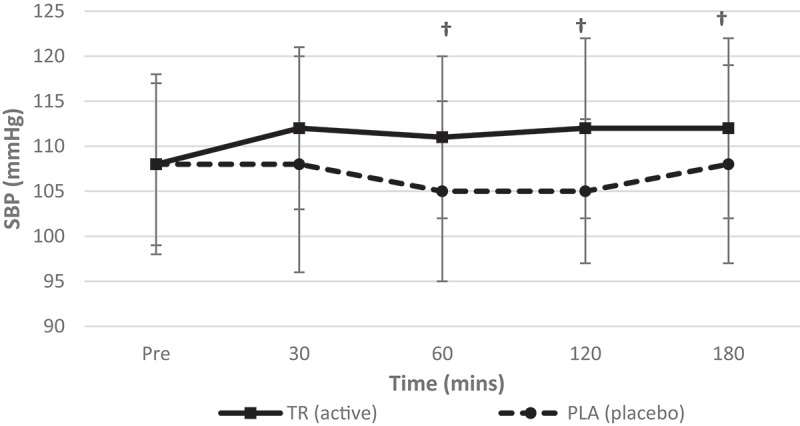
Systolic blood pressure over time. There was no main effect for time; however, there was an observed significant interaction (condition*time) and a main effect for condition for SBP. There were also significant differences between conditions at 60, 120, and 180 min post ingestion (condition: TR = active; PLA = placebo). †Denotes statistical significance at *p* < 0.05 for differences between conditions.

A significant condition*time interaction (*F* = 3.156, *p* = 0.019, η_p_^2^ = 0.131) as well as a main effect for condition (*F* = 10.367, *p* = 0.004, η_p_^2^ = 0.330) was observed for SBP (Figure 7). There was no observed main effect for time (*F* = 1.110, *p* = 0.355, η_p_^2^ = 0.050) for SBP. Furthermore, there were observed significant differences for SBP between conditions at 60, 120, and 180 min post ingestion (*p* = 0.005, *p* = 0.001, *p* = 0.049, respectively).

**Figure 8.**
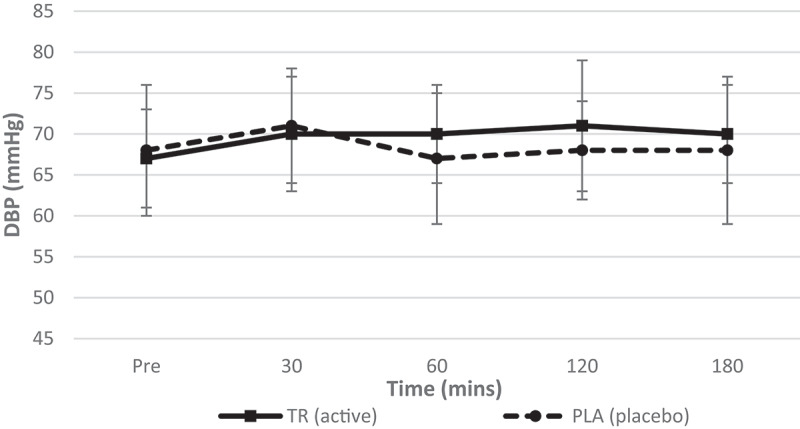
Diastolic blood pressure over time. There was no significant interaction (condition*time) or main effects for time and condition for DBP.

There were no significant condition*time interactions (*F* = 0.729, *p* = 0.464, η_p_^2^ = 0.153), main effects for time (*F* = 1.022, *p* = 0.370, η_p_^2^ = 0.046), or condition (*F* = 0.854, *p* = 0.366, η_p_^2^ = 0.039) for diastolic blood pressure (DBP) (Figure 8).

### Subjective mood states

No significant interaction (condition*time) was observed (*F* = 1.910, *p* = 0.153, η_p_^2^ = 0.080) for hunger and satiety. While a significant main effect for time (*F* = 11.138, *p* = 0.000, η_p_^2^ = 0.336) was observed, no main effect for condition (*F* = 0.230, *p* = 0.636, η_p_^2^ = 0.010) was observed. Post-hoc testing revealed significant differences from baseline to 120 and 180 min post ingestion (*p* = 0.002, *p* <0.001, respectively) and from baseline to 180 min for PLA (*p* = 0.027).

No significant interaction (condition*time) was observed (*F* = 0.987, *p* = 0.399, η_p_^2^ = 0.043) for subjective perceived energy levels. A significant main effect for time (*F* = 7.427, *p* = 0.001, η_p_^2^ = 0.252) was observed, but not for condition (*F* = 4.069, *p* = 0.056, η_p_^2^ = 0.156), was observed. Post-hoc testing revealed significant differences from baseline to 30, 60, 120, and 180 min (*p* = 0.002, *p* = 0.003, *p* = 0.036, *p* = 0.006, respectively).

A significant condition*time interaction was observed (*F* = 3.042, *p* = 0.037, η_p_^2^ = 0.121) for subjective levels of focus. There was a significant main effect for time (*F* = 8.043, *p* < 0.001, η_p_^2^ = 0.268) but not condition (*F* = 0.467, *p* = 0.502, η_p_^2^ = 0.021). Post-hoc testing further revealed significant differences in TR from baseline to 30, 60, 120, and 180 min post ingestion (*p* = 0.009, *p* < 0.001, *p* = 0.002, *p* = 0.001, respectively), while in PLA the only differences were found from baseline to 180 min post ingestion (*p* = 0.038).

No significant interaction (condition*time) was observed (*F* = 1.177, *p* = 0.325, η_p_^2^ = 0.051) for subjective perceived levels of concentration. A significant main effect for time (*F* = 6.090, *p* = 0.002, η_p_^2^ = 0.217), but not condition (*F* = 1.033, *p* = 0.321, η_p_^2^ = 0.045), was observed. Post-hoc testing revealed significant differences from baseline to 60, 120, and 180 min (*p* = 0.001, *p* = 0.015, *p* = 0.002, respectively).

No significant interaction (condition*time) was observed (*F* = 1.645, *p* = 0.187, η_p_^2^ = 0.070) for subjective levels of alertness. A significant main effect for time (*F* = 6.911, *p* = 0.002, η_p_^2^ = 0.239), but not condition (*F* = 2.335, *p* = 0.141, η_p_^2^ = 0.096), was observed. Post-hoc testing showed significant differences from baseline to 30, 120, and 180 min post ingestion (*p* = 0.002, *p* = 0.038, *p* = 0.012, respectively).

No significant interaction (condition*time) (*F* = 2.581, *p* = 0.060, η_p_^2^ = 0.105) or main effects for time (*F* = 1.020, *p* = 0.378, η_p_^2^ = 0.044) or condition (*F* = 1.442, *p* = 0.243, η_p_^2^ = 0.062) observed for subjective levels of fatigue.

### Blood variables

**Figure 9.**
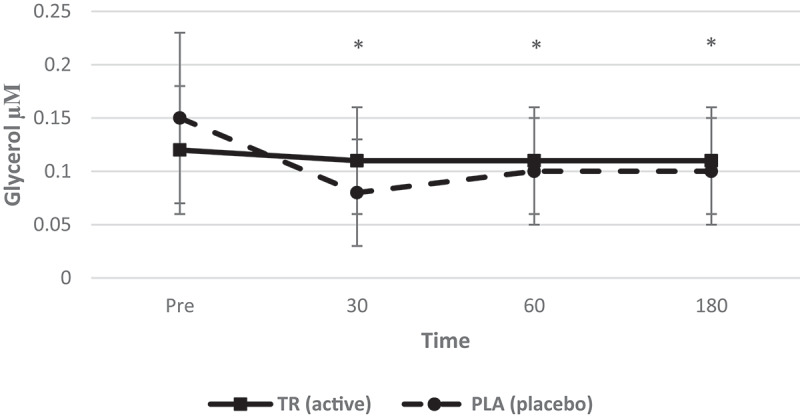
Glycerol concentration over time. A significant interaction (condition*time) and main effect for time for serum glycerol. Glycerol levels were maintained post ingestion of TR, while there was a decrease of glycerol at 30, 60, and 180 min post-ingestion of PLA (condition: TR = active; PLA = placebo. *Denotes statistical significance at *p* < 0.05 for differences from baseline to each timepoint.

**Figure 10.**
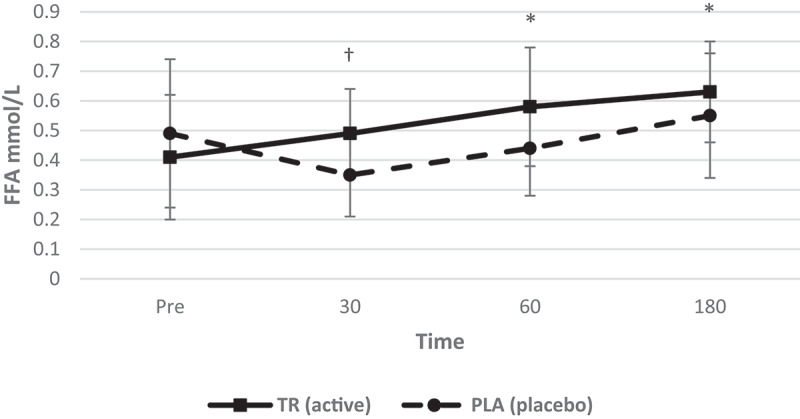
Free fatty acid concentration over time. A significant interaction (condition*time) as well as main effects for condition and time were observed. Free fatty acid levels increased at 60 and 180 min post-ingestion of TR and free fatty acid levels were greater than PLA at 30 min (condition: 1 = TR; 2 = PLA). *Denotes statistical significance at *p* < 0.05 for differences from baseline to each timepoint; †denotes statistical significance at *p* < 0.05 for differences between conditions.

Glycerol (Figure 9) and free fatty acids (Figure 10) were assessed for changes in concentration at multiple timepoints over the course of each 3 hr testing session. There was a significant condition*time interaction for glycerol (*F* = 6.653, *p* = 0.001, η_p_^2^ = 0.241). There was also a significant main effect for time (*F* = 8.573, *p* < 0.001, η_p_^2^ = 0.290) but not condition (*F* = 0.159, *p* = 0.694, η_p_^2^ = 0.008). Fasting glycerol levels were maintained over 3 h in the TR group, while glycerol decreased from baseline to 30 (*p* = 0.0013), 60 (*p* = 0.0286), and 180 min (*p* = 0.0390) post-ingestion in the PLA group.

There was also a statistically significant condition*time interaction for free fatty acids (*F* = 10.994, *p* < 0.001, η_p_^2^ = 0.355). There was also a significant main effect for time (*F* = 18.055, *p* < 0.001, η_p_^2^ = 0.474) and condition (*F* = 4.343, *p* = 0.050, η_p_^2^ = 0.178). Free fatty acid levels significantly increased in TR from baseline to 60 min (*p* = 0.0299) and 180 min (*p* = 0.0007), as well as a significant difference between treatments at 30 min (*p* = 0.0095) with TR having a higher serum concentration of free fatty acids post-ingestion.

## Discussion

The key findings from this experiment indicated that the ingestion of a specific thermogenic supplement formulation produces a sustained elevation of metabolic rate and caloric expenditure, increases serum-free fatty acid levels, prevents a decrease in fasting glycerol levels, and increases focus over the 3 h testing session when compared to PL without producing adverse hemodynamic responses.

Caffeine is absorbed rapidly through the gastrointestinal tract and is metabolized in the liver before it crosses the blood–brain barrier and affects neural function [9]. Caffeine mimics the sympathetic nervous system and leads to an increased release of catecholamines, epinephrine, and norepinephrine [11]. Epinephrine binds to the beta-receptors in adipocytes. This activates protein kinase-A, stimulates the activity of hormone-sensitive lipase, and helps degrade triglycerides and mobilize free fatty acids and glycerol via hydrolysis [12]. The ability of caffeine to stimulate SNS activity, and thus, thermogenesis, is due to its ability to inhibit PDE and allow the accumulation of cAMP, as well as induce an increase in lipolysis, which makes caffeine a primary ingredient in many thermogenic supplements. Caffeine alone has been shown to increase metabolic rate in trained and untrained males and females at various moderate doses [13–20]. Similar to the findings of the present study, multiple studies have demonstrated that metabolic rate was moderately increased in both normal weight [13,14,19] and clinically obese [14,20] women for a short period. The 150 mg dose in the present study was smaller than the aforementioned experiments. This data can add to the current literature for caffeine’s effects on metabolic rate at a lower acute dose of caffeine.

Combining caffeine with other thermogenic or herbal ingredients such as L-carnitine, green tea leaf extract, guarana seed extract, and others have also been shown to induce a thermogenic effect and elevate REE similar to the present findings [3,4,6,7,15,21,22]. The main ingredients in this thermogenic supplement’s “fat burning matrix” (containing 2003 mg of various ingredients) are acetyl L-carnitine HCl, *Garcinia cambogia* fruit extract (60% hydroxycitric acid), conjugated linoleic acid, and bitter orange (p-synephrine), which all have less research regarding their efficacy concerning their effects on metabolic rate and mobilization of stored fat when compared to caffeine. Additionally, the inclusion of bitter orange (p-synephrine) in this blend should be noted as well as an acute stimulator of metabolism.

Bitter orange (p-synephrine) has a chemical structure that is similar to ephedrine, and its proposed mechanism of action in humans involves adipogenesis-related protein suppression [CCAAT-enhancer-binding protein α (C/EBPα) and peroxisome proliferator-activated receptor γ (PPARγ)] [23]. Furthermore, calcium and cAMP signaling pathways and targeting adrenergic receptors [24] are also related to the anti-adipogenic effect of p-synephrine. Bitter orange extract has been deemed safe for humans to consume both alone and combined with caffeine at rest [25] as well as during exercise [26]. Some research has reported that bitter orange can acutely increase fat oxidation at rest [27] and during or following exercise [27–29] without producing harmful cardiovascular effects, which makes it a promising ingredient in thermogenic supplements.

Acetyl L-carnitine is a supplemental form of L-carnitine that is theorized to influence the transportation of fatty acids across the mitochondrial membrane in the process of fat metabolism [30]. A recent meta-analysis [31] reviewed the literature to date on the effects of L-carnitine on weight loss and body composition and found that doses up to 2000 mg per day with chronic supplementation can reduce body weight, BMI, and fat mass, but not waist circumference or body fat percent, particularly in clinically overweight or obese males and females. Despite the limited data on acute ingestion of L-carnitine on measures of metabolic rate, longitudinal studies discussed above do provide rationale for being an active ingredient in products similar to the ones investigated in this trial. Future research to explore the thermogenesis of acetyl L-carnitine alone would substantiate these claims.

A review of the efficacy of *Garcinia cambogia* fruit extract (GCFE), another popular ingredient in weight loss products, highlights that this ingredient has contradicting data regarding its effect on body composition. One study [32] and two systematic reviews [33,34] highlight the lack of significant positive effects on resting energy expenditure, weight loss, or satiety in overweight or obese males and females between 20 and 70 years of age after chronic supplementation of 2–5 g/day of GCFE. Another systematic review [35] concluded that there may be a small magnitude of effect for GCFE with short-term weight loss with 1–3 g chronic supplementation. Since the research available is limited and contradicting and mostly concentrated on chronic supplementation, the influence of acute ingestion of GCFE on metabolic rate and related variables should be explored.

The effects of conjugated linoleic acid (CLA) on body composition and metabolism were reviewed [36]. Although there are no acute trials that examine the effects of CLA on metabolic rate, there are some studies that have shown that CLA can reduce fat mass and alter body composition [37,38] in males and females; however, there are also studies that have shown no significant differences between CLA and placebo [39,40]. An increase in metabolic rate was observed following 13 weeks of CLA supplementation [41], which contradicts previous findings that state that there were no observed differences between CLA and PLA with chronic supplementation [42]. Although the effects of long-term ingestion of this product and its ingredients like CLA was not evaluated in this trial, data showing an increase in metabolic rate when consumed over time does provide some evidence for justifying the inclusion of CLA in a thermogenic/weight loss blended dietary supplement.

Markers of fat metabolism in the blood stream analyzed here included an observed increased level of free fatty acids and sustained glycerol following the ingestion of the experimental treatment, which could be activated by acute caffeine ingestion. The inhibition of PDE and accumulation of cAMP that is caused by caffeine leads to an increase in circulating free fatty acids that may then be used as fuel for the body. The sympathomimetic property of caffeine also leads to the degradation of triglycerides, thereby mobilizing free fatty acids and glycerol. However, with the acute dose and prescribed resting conditions in the present study, it is unclear whether the mobilization was significant enough to drive fat oxidation as indicated by the observed changes in RQ over time.

Hemodynamic responses such as heart rate and blood pressure were also altered, but the magnitude of change was not large enough to raise concern as all values were within normal ranges. Increases in SBP were observed (4 mmHg) but again within normal ranges, and there were no observed changes to DBP. These observations indicate that there were no adverse hemodynamic responses after ingesting an acute dose of this specific thermogenic supplement. The observed changes in SBP, DBP, and HR were mild and consistent with the current literature available on acute doses of caffeine up to 600 mg in males and females [43].

The overall increase in subjective levels of focus supports similar research in females [44] in which there were comparable findings upon ingestion of a multi-ingredient thermogenic supplement containing caffeine. There were main effects for time observed for subjective, energy, concentration, and alertness; however, without a significant condition by time interaction or main effect for condition we cannot attribute these changes to the thermogenic supplement, coinciding with other findings in females [44]. These findings corroborate other similar research in males and females [45] where main effects were present in the absence of a statistical interaction for similar subjective variables. The lack of changes observed for subjective fatigue is contradictory to similar research in males and females [46,47], which observed decreases in fatigue after ingesting a multi-ingredient supplement containing caffeine in males and females.

## Conclusion

The major findings of this study indicate that ingesting a commercially available thermogenic supplement formulation (OxyShred), containing 150 mg of caffeine and 2003 mg of a “fat-burning matrix” that consists of various ingredients including bitter orange (p-synephrine), acetyl L-carnitine HCl, *Garcinia cambogia* fruit extract, and conjugated linoleic acid, can produce a sustained, significant increase metabolic rate, significant increase in circulating free fatty acids, and increased focus over a 3 h time period when compared to placebo without producing adverse hemodynamic responses or other measured adverse events. Collectively the ingredients highlighted above individually have research that supports their role as being an active ingredient in products such as the one investigated here. However, since this trial studied the combined ingestion of this thermogenic dietary supplement we cannot speculate further on their individual effects as a result of this trial. It is unknown whether daily consumption of this supplement will continue to produce elevated REE values, or whether it will contribute to weight loss, fat loss, or weight maintenance. Further research should investigate the efficacy of ingesting the thermogenic supplement over a longer period of time.
